# Association between holiday and weekend admissions and mortality outcomes among patients with acute myocardial infarction receiving percutaneous coronary intervention in Taiwan

**DOI:** 10.1038/s41598-024-59571-w

**Published:** 2024-04-17

**Authors:** Peter Pin-Sung Liu, Huai-Ren Chang, Jin-Yi Hsu, Huei-Kai Huang, Ching-Hui Loh, Jih-I Yeh

**Affiliations:** 1 Institute of Medical Sciences, Tzu Chi University, Hualien, Taiwan; 2 Center for Aging and Health, Hualien Tzu Chi Hospital, Buddhist Tzu Chi Medical Foundation, Hualien, Taiwan; 3 School of Medicine, Tzu Chi University, Hualien, Taiwan; 4 Division of Cardiology, Department of Internal Medicine, Hualien Tzu Chi Hospital, Buddhist Tzu Chi Medical Foundation, Hualien, Taiwan; 5 Department of Family Medicine, Hualien Tzu Chi Hospital, Buddhist Tzu Chi Medical Foundation, No. 707, Sec. 3, Chung Yang Rd., Hualien, 97002 Taiwan; 6 Department of Medical Research, Hualien Tzu Chi Hospital, Buddhist Tzu Chi Medical Foundation, Hualien, Taiwan; 7 Center for Healthy Longevity, Hualien Tzu Chi Hospital, Buddhist Tzu Chi Medical Foundation, Hualien, Taiwan

**Keywords:** Myocardial infarction, Mortality, Holiday season, Weekend effect, Cardiology, Epidemiology, Health care, Health services, Public health

## Abstract

There is a lack of studies that concurrently differentiate the effect of the holiday season from the weekend effect on mortality risk in patients with acute myocardial infarction (AMI). We evaluated the mortality risk among patients admitted with AMI who underwent percutaneous coronary intervention, using data from the Taiwan National Health Insurance Research Database. Adult AMI patients admitted during January and February between 2013 and 2020 were enrolled and classified into the holiday season (using the Chinese New Year holiday seasons as an indicator) (n = 1729), weekend (n = 4725), and weekday (n = 14,583) groups according to the first day of admission. A multivariable logistic regression model was used to assess the risk. With the weekday group or the weekend group as the reference, the holiday season group did not have increased risks of in-hospital mortality (adjusted odds ratio [aOR] 1.15; 95% confidence intervals [CI] 0.93–1.42 or aOR 1.23; 95% CI 0.96–1.56) and 7-day mortality (aOR 1.20; 95% CI 0.90–1.58 or aOR 1.24; 95% CI 0.90–1.70). Stratified and subgroup analyses showed similar trends. We conclude that holiday season-initiated admissions were not associated with higher mortality risks in AMI admission cases than weekday or weekend admissions.

## Introduction

Acute myocardial infarction (AMI) is a life-threatening condition worldwide^1^. Previous studies have shown that the mortality rate of patients with AMI admitted to hospitals on weekends is higher than that of patients admitted on weekdays. This phenomenon has been termed as the “weekend effect”^2,3^. Prior studies have also identified potential problems underlying the “weekend effect”. These include prolonged time from presentation to diagnosis^4^, severity of the disease^5^, disparities in the levels of staffing between weekends and weekdays^6^, and accessibility of primary percutaneous coronary intervention (PCI)^7^.

Holiday seasons are important for healthcare staff to take vacations and engage with their families. A shortage of healthcare professionals could be a more crucial problem during holiday seasons than on weekends, especially during holiday seasons that last longer than three days. This situation suggests that patients admitted during the holiday season may have worse outcomes than those admitted during regular times. Several previous studies on AMI patients combined holiday season admissions with weekend admissions^8–10^; however, studies specifically evaluating the effect of holiday season admissions on the prognosis of AMI patients are limited^11^.

To date, no study had concurrently differentiated the effect of the holiday season from the weekend effect on mortality risk in patients with AMI. Understanding such correlations may help in efforts toward improving public health strategies, medical resource allocation, and workforce scheduling, consequently improving the quality of outcomes in patients with AMI. Thus, we investigated the association of holiday season, weekend, and weekday admissions with mortality risk in patients admitted for AMI receiving PCI.

## Materials and methods

### Data sources

The current study was structured in accordance with the Reporting of Studies Conducted using Observational Routinely-Collected Data (RECORD) guidelines, and we stated the required items in an online appendix^12^.

We conducted this study using data from the Taiwan National Health Insurance Research Database (NHIRD). Taiwan is an East Asian democratic country with 99% of its citizens (~ 23.6 million) enrolled in the National Health Insurance (NHI) programme launched in 1995. The NHI is a single-payer, universal-coverage health insurance system, providing comprehensive health services, including outpatient visits, inpatient admissions, emergency department services, and pharmacy prescriptions. Taiwan’s Ministry of Health and Welfare manages the database in the Health and Welfare Data Centre (HWDC). The investigator who received the approval to access the data performed analysis the data in an isolated computer room of the HWDC to ensure privacy and safety of the population’s information. Besides the NHIRD, Taiwan’s National Death Registry could also be accessed by the researchers, making the mortality record precise^13^. Our study was approved by the Tzu Chi General Hospital Research Ethics Committee (approval number IRB107-152-C). The requirement for informed consent was waived owing to the retrospective study design.

### Study population and classifications

This study included adult patients (aged ≥ 20 years) who were hospitalized with an AMI diagnosis and underwent PCI between 2013 and 2020 in January and February. We defined AMI using the International Classification of Diseases, Ninth Revision, Clinical Modification (ICD-9-CM) codes 410, and the International Classification of Diseases, Tenth Revision, Clinical Modification (ICD-10-CM) codes I21. PCI was defined according to the NHI billing code (Table S1). A previous study that examined the agreement of these diagnosis codes with clinical data reported a positive predictive value of 0.88, confirming the accuracy^14^. The hospital admission date was used as the index date.

In this study, the “holiday season” indicated the Chinese New Year, a festival with at least four consecutive holidays (during January and February) in Taiwan and the duration occasionally extends into the adjoining weekend. Government offices, schools, and businesses remained closed during the Chinese New Year. Healthcare system agencies reduce their staffing and capacity to a level that is lower than that during weekends. We extracted the exact dates of the Chinese New Year from Taiwan’s official calendar (Table S2), and enrolled AMI admissions that occurred in January and February to minimize the influence of seasonal variations on the calculated mortality rates. Other national holidays in January or February were categorized as weekdays or weekends depending on which day of the week they fall on. The patients were classified into the holiday season, weekend, and weekday groups when their index dates were during the Chinese New Year holiday season, weekends (Saturday and Sunday), and weekdays (Monday to Friday), respectively. We compared the mortality outcomes among these groups at in-hospital and 7-day of index dates, using weekdays as the reference group. Furthermore, we compared the mortality outcomes between the holiday season and weekend groups, using the weekend as the reference group.

Some patients were also excluded from the study. As our investigation focused on new-onset AMI admission, patients with any AMI diagnosis who had an outpatient or inpatient visit before the index date were excluded. Patients admitted to the remote island hospitals were also excluded because the equipment and transfer model are disparate from those of mainland Taiwan. Comprehensive treatment facilities for patients with AMI exist in medical centers or regional hospitals. However, a subset of patients who underwent treatment at district hospitals was excluded because of the severity of AMI and treatment protocol in these patients might have differed from those in our target population.

### Study outcomes and follow-up

The primary outcome was all-cause mortality, evaluated for the in-hospital admission period and 7-day after the index date and was determined using the National Death Registry.

### Covariates and potential confounders

Patient baseline characteristics, including demographic factors, AMI type, comorbidities, and hospital admission characteristics, were collected. The monthly income-related insurance premiums were categorized into four levels (New Taiwan dollars ≥ 45,000, 25,000–44,999, 15,840–24,999, and financially dependent [< 15,840, the minimum wage]). The index year was defined as the year of the index date. Physician were classified as cardiologists, cardiothoracic surgeons, or others. Transfer-in admission was defined as any emergency department visit record with an AMI diagnosis from another hospital within one day of the index date. Out-of-hospital cardiac arrest (OHCA) was defined using the diagnostic code of cardiac arrest (ICD-9-CM code: 427.5; ICD-10-CM code: I46.9) in the emergency department^15,16^. We identified ST-elevation myocardial infarction (STEMI) using ICD-9-CM codes (410.0-6, 410.8) and ICD-10-CM codes (I21.0-3). Comorbidities were identified by analyzing the medical visit records from one year before the index date. A preexisting comorbidity was defined as a disease diagnosed during at least one inpatient or two outpatient visits in the year preceding the index date. Charlson’s Comorbidity Index scores were calculated using the algorithm described before^17^. The geographic locations of the admission hospitals were separated into four areas: north, central, south, and east. Hospital levels were certified by the Taiwan Joint Commission on Hospital Accreditation; the levels were: medical center, regional hospital, and district hospital, determined by their healthcare capabilities, quality of outcomes, bed size, and teaching abilities of the hospital. In this study, we only included the admissions of medical centers or regional hospitals. Patients who received a coronary artery bypass graft (CABG) or were admitted to the intensive care unit (ICU) were defined using NHI claim codes. (Table S2).

### Stratified and subgroup analysis

Stratified analyses were performed according to sex (male or female), age (< 65 years or ≥ 65 years), and AMI types (NSTEMI or STEMI). We conducted subgroup analyses after excluding patients who had OHCA, received CABG, and underwent inter-hospital transfer, as the disease severity and delayed timing could bias the observational results.

### Statistical analysis

The baseline characteristics were addressed using number (percentage) for categorical variables and mean (standard deviation [SD]) for numerical variables. The differences between three groups were assessed using independent *t*-test for numerical variable or χ^2^ test for categorical variable. We also applied the standardized mean difference (SMD) to quantify the difference at baseline between groups because the *P* value could be influenced by a large sample size; if the value of SMD < 0.1 was considered negligible^18,19^. A multivariable logistic regression model was employed to obtain the adjusted odds ratio (aOR) for the mortality event. The aORs and corresponding 95% CI were reported after adjusting for all covariates listed in Table 1.

**Table 1:** Baseline characteristics of patients admitted during holiday seasons, weekends, and weekdays.

Variable	Holiday season n = 1729	Weekend n = 4725	Weekday n = 14,583	P value^#^
Age* (yeas)	66.0 (13.7)	66.0 (13.9)	65.9 (13.8)	0.798
< 65	784 (45.3)	2186 (46.3)	6764 (46.4)	0.715
≥ 65	945 (54.7)	2539 (53.7)	7819 (53.6)	
Sex				
Male	1320 (76.3)	3551 (75.2)	10,901 (74.8)	0.333
Female	409 (23.7)	1174 (24.8)	3682 (25.2)	
Insurance premium level				
Financially dependent	485 (28.1)	1242 (26.3)	3967 (27.2)	0.078
15,840–24,999	686 (39.7)	2064 (43.7)	6305 (43.2)	
25,000–44,999	299 (17.3)	782 (16.6)	2329 (16.0)	
≥ 45,000	259 (15.0)	637 (13.5)	1982 (13.6)	
Hospital level				
Medical center	827 (47.8)	2092 (44.3)	6214 (42.6)	< 0.001
Regional hospital	902 (52.2)	2633 (55.7)	8369 (57.4)	
Hospital area location				
North	705 (40.8)	2066 (43.7)	6336 (43.4)	0.204
Central	372 (21.5)	1027 (21.7)	3057 (21.0)	
South	595 (34.4)	1504 (31.8)	4745 (32.5)	
East	57 (3.3)	128 (2.7)	445 (3.1)	
Physician type				
Cardiologist	1561 (90.3)	4235 (89.6)	13,050 (89.5)	0.687
Cardiothoracic surgeon	79 (4.6)	249 (5.3)	739 (5.1)	
Other	89 (5.1)	241 (5.1)	794 (5.4)	
AMI type				
STEMI	749 (43.3)	1976 (41.8)	5886 (40.4)	0.023
NSTEMI	980 (56.7)	2749 (58.2)	8697 (59.6)	
Transferred-in	275 (15.9)	703 (14.9)	1702 (11.7)	< 0.001
OHCA	45 (2.6)	135 (2.9)	332 (2.3)	0.071
CABG	69 (4.0)	208 (4.4)	667 (4.6)	0.514
ICU	1574 (91.0)	4331 (91.7)	13,114 (89.9)	0.001
Length of stay*	9.1 (10.6)	9.1 (11.5)	9.1 (11.7)	0.936
CCI score*	1.6 (2.0)	1.6 (2.0)	1.7 (2.1)	0.032
Comorbidities				
DM	627 (36.3)	1673 (35.4)	5406 (37.1)	0.113
HTN	893 (51.6)	2537 (53.7)	7860 (53.9)	0.207
COPD	112 (6.5)	368 (7.8)	1068 (7.3)	0.195
Heart failure	111 (6.4)	341 (7.2)	1173 (8.0)	0.019
CAD	330 (19.1)	964 (20.4)	3132 (21.5)	0.033
CKD	293 (16.9)	692 (14.6)	2469 (16.9)	0.001
Cirrhosis	33 (1.9)	119 (2.5)	359 (2.5)	0.333
Stroke	178 (10.3)	518 (11.0)	1570 (10.8)	0.745
Dementia	55 (3.2)	130 (2.8)	483 (3.3)	0.161
Malignancy	88 (5.1)	245 (5.2)	745 (5.1)	0.976
Index year				
2013	135 (7.8)	544 (11.5)	1613 (11.1)	< 0.001
2014	147 (8.5)	548 (11.6)	1818 (12.5)	
2015	201 (11.6)	543 (11.5)	1726 (11.8)	
2016	185 (10.7)	706 (14.9)	1841 (12.6)	
2017	193 (11.2)	651 (13.8)	1923 (13.2)	
2018	301 (17.4)	619 (13.1)	1845 (12.7)	
2019	395 (22.8)	469 (9.9)	1864 (12.8)	
2020	172 (9.9)	645 (13.7)	1953 (13.4)	

*AMI* acute myocardial infarction, *STEMI* ST-elevation myocardial infarction, *NSTEMI* non-ST-elevation myocardial infarction, *OHCA* out-of-hospital cardiac arrest, *CABG* coronary artery bypass graft, *ICU* intensive care unit, *CCI* Charlson comorbidity index, *DM* diabetes mellitus, *HTN* hypertension, *COPD* chronic obstructive pulmonary disease, *CAD* coronary arterial disease, *CKD* chronic kidney disease.

*Expressed as mean and SD.

^#^Significant test p value using one-way ANOVA for numerical variable or χ^2^ test for categorical variable.

Significance (two-tailed) was set at *P* < 0.05. Statistical analyses were performed using SAS software, version 9.4 (SAS Institute, Inc., Cary, NC, USA) and R software, version 4.1.2 (R Foundation for Statistical Computing, Vienna, Austria).

### Ethics approval and consent to participate

This study was conducted in accordance with the Declaration of Helsinki. The study protocol was reviewed and approved by the Research Ethics Committee of the Buddhist Tzu Chi Medical Foundation. Our study was approved with a waiver of the requirement for informed consent by the Research Ethics Committee of the Buddhist Tzu Chi Medical Foundation (approval number IRB110-170-C).

### Declaration of generative AI and AI-assisted technologies in the writing process

During the preparation of this work the authors used Chat GPT v.3.5 (Open AI, 2023) in order to check grammar. After using this tool/service, the authors reviewed and edited the content as needed and take full responsibility for the content of the publication.

## Results

### Patient characteristics

A total of 21,037 patients with AMI were hospitalized receiving PCI during the study period; 1729, 4725, and 14,583 admissions were categorized into the holiday season, weekend, and weekday groups, respectively. The distribution (mean [years] ± SD) of age (holiday season: 66.0 ± 13.7; weekend: 66.0 ± 13.9; weekday: 65.9 ± 13.8) and the proportion of sexes (holiday season: male 76.3%; weekend: male 75.2%; weekday: male 74.8%) were similar among the three groups. Table 1 shows the patients’ baseline characteristics and the comparison results using independent *t*-test or χ^2^ test. There were some differences in AMI type, transferred patients, admitted in ICU, CCI score, history of heart failure, CAD, and CKD between three groups (Table 1). To avoid the issue of p value for hypothesis test in big data, we also provide the pairwise comparison results using SMD in Table S3. Although most characteristics were similar among the three groups, the holiday season group showed a slightly higher proportion of transfers from other hospitals’ emergency departments than the weekday group (15.9% vs. 11.7%, SMD = 0.122) (Table S3).

### Mortality risks

In total, there were 1335 (6.3%) and 688 (3.3%) mortality events within the in-hospital and 7-day periods, respectively. The age-sex standardized 30-day mortality rate for patients with AMI aged 45 years of our patients during the study period was 4.9%. The mortality rates and the adjusted odds ratios (aOR) with 95% CI are reported in Table 2. Patients admitted for AMI who underwent PCI during the holiday season had a similar mortality risk to those with weekday-initial or weekend-initial admission on multivariable analyses after adjusting for the covariates listed in Table 1. The numbers of admissions of AMI patients per day in 2019, 2018, 2017, and 2016 were 44, 50, 48, and 46, respectively. There were no differences observed among the holiday, weekend, and weekday groups. Detailed daily case numbers are presented in Table S4. Subgroup analysis according to hospital levels revealed that the 7-day mortality on weekdays, not on weekends or holiday seasons, in regional hospitals was significantly higher than in medical centers (Table S5). Also, subgroup analysis according to geographic regions revealed that the 7-day mortality during the holiday season, not on weekdays or weekends, in northern Taiwan was significantly higher than in other regions (Table S5). No significant differences in in-hospital mortality were found among hospital levels or geographic regions (Tables S5 and S6).

**Table 2:** Mortality risks in patients with AMI during the holiday seasons, weekends, and weekdays.

Mortality type	Date category	N	Mortality events	Mortality rate (%)	crude ORs* (95% CI)	*P* value	aORs^†^ (95% CI)	*P* value	crude ORs^‡^ (95% CI)	*P* value	aORs^§^ (95% CI)	*P* value
In-hospital mortality	Holiday season	1729	119	6.9	1.10 (0.90–1.33)	0.350	1.15 (0.93–1.42)	0.198	1.10 (0.88–1.37)	0.387	1.23 (0.96–1.56)	0.094
	Weekend	4725	297	6.3	1.00 (0.87–1.14)	0.968	0.96 (0.83–1.11)	0.576	1.00 (ref.)		1.00 (ref.)	
	Weekday	14,583	919	6.3	1.00 (ref.)		1.00 (ref.)		–		–	
7-day mortality	Holiday season	1729	64	3.7	1.17 (0.89–1.51)	0.255	1.20 (0.90–1.58)	0.197	1.10 (0.82–1.48)	0.512	1.24 (0.90–1.70)	0.182
	Weekend	4725	159	3.4	1.06 (0.88–1.27)	0.551	1.00 (0.82–1.20)	0.974	1.00 (ref.)		1.00 (ref.)	
	Weekday	14,583	465	3.2	1.00 (ref.)		1.00 (ref.)		–		–	

*The OR is calculated by univariable logistic regression model using weekday as reference group.

^†^The OR is calculated by multivariable logistic regression model with adjustments for the characteristics listed in Table 1 using weekday as reference group.

^‡^The OR is calculated by univariable logistic regression model using weekend as reference group.

^§^The OR is calculated by multivariable logistic regression model with adjustments for the characteristics listed in Table 1 using weekend as reference group.

*AMI* acute myocardial infarction, *aOR* adjusted odds ratio, *CI* confidence interval, *ref* reference group.

There was no significant excess risk in the holiday season group when compared with the weekday group for in-hospital mortality (aOR 1.15; 95% CI 0.93–1.42, *P* = 0.198) and 7-day mortality (aOR 1.20; 95% CI 0.90–1.58, *P* = 0.197) (Table 2). Furthermore, we compared the mortality risk between the holiday season group and weekend group using weekend group as reference. The aOR was 1.23 (95% CI 0.96–1.56, *P* = 0.094) for in-hospital mortality and 1.24 (95% CI 0.90–1.70, *P* = 0.182) (Table 2).

### Stratified analysis by age, sex, and AMI type

**Figure 1:**
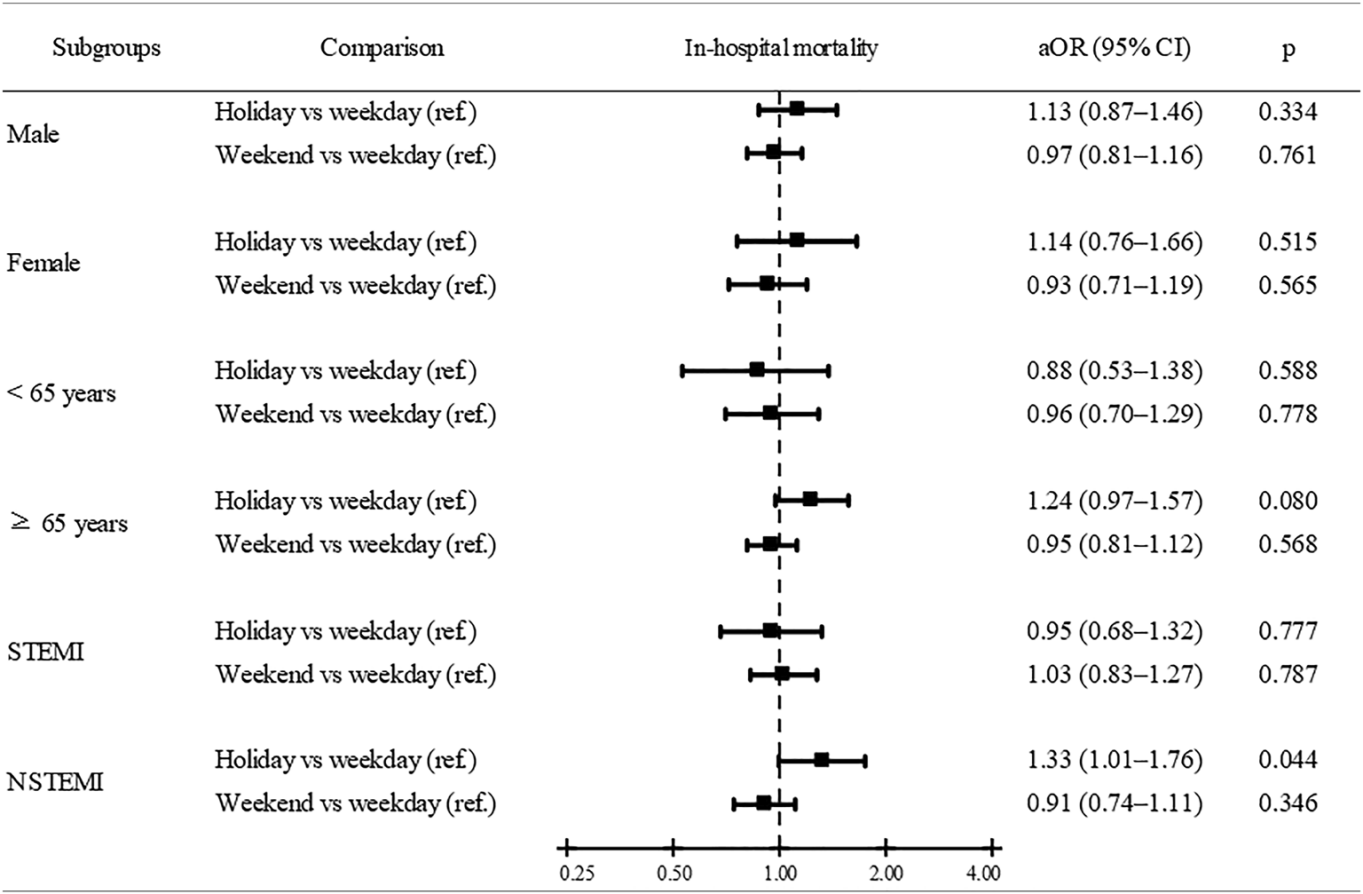
In-hospital mortality risks in patients with AMI during the holiday seasons, weekends, and weekdays stratified by sex, age group, and AMI type. *AMI* acute myocardial infarction, *aOR* adjusted odds ratio, *CI* confidence interval, *ref* reference group, *STEMI* ST-elevation myocardial infarction, *NSTEMI* non-ST-elevation myocardial infarction.

The stratified analysis, including age, sex, and AMI type stratification, revealed similar trends along with the main findings (Figs. 1 and S1). However, the result in the NSTEMI subgroup showed that the patients admitted during the holiday season had a higher mortality risk than those in the weekday admission groups. The aOR was 1.33 (95% CI 1.01–1.76, *P* = 0.044) for in-hospital mortality (Fig. 1).

### Subgroup analysis

**Figure 2:**
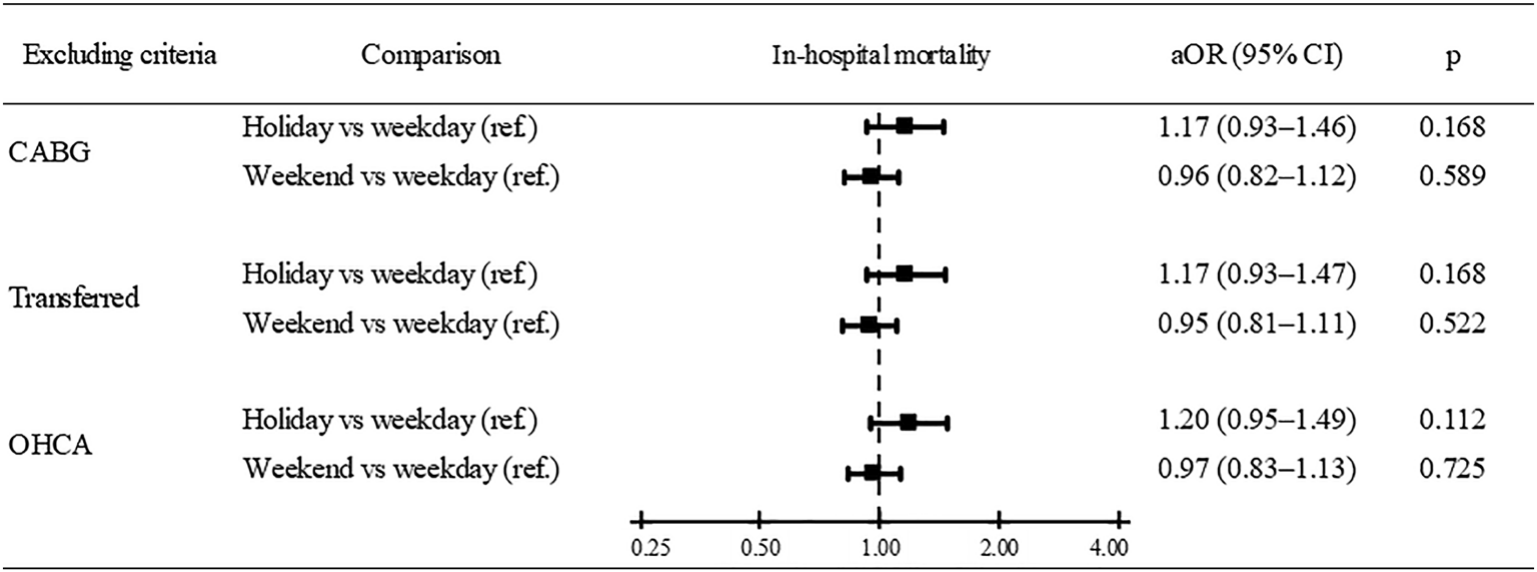
Sensitivity analysis for in-hospital mortality risk after excluding patients underwent CABG, transferred, and OHCA. *aOR* adjusted odds ratio, *CI* confidence interval, *ref* reference group, *CABG* coronary artery bypass graft, *OHCA* out-of-hospital cardiac arrest.

We excluded the patients who had OHCA, received CABG, or underwent inter-hospital transfer to perform additional subgroup analyses. Figure 2 showed that the patients admitted during the holiday season had no higher in-hospital mortality risk than those in the weekday admission groups. The aOR for in-hospital mortality outcome was 1.17 (95% CI 0.93–1.47, *P* = 0.168) for subgroup excluding OHCA, 1.17 (95% CI 0.93–1.46, *P* = 0.168) for subgroup excluding CABG, and 1.20 (95% CI 0.83–1.13, *P* = 0.725) for subgroup excluding transfer (Fig. 2). The subgroup analyses for 7-day outcomes revealed similar trends (Fig. S2).

## Discussion

Our study showed that patients admitted for AMI receiving PCI during holiday seasons and weekends did not have a significantly higher mortality risk than those admitted during weekdays in terms of in-hospital and 7-day mortality outcomes. These patterns remained consistent in the stratified and subgroup analyses. To the best of our knowledge, this is the first study to investigate the effect of the “holiday season” in AMI inpatients’ mortality.

In the subgroup analysis of NSTEMI, the in-hospital mortality rate was associated with admissions during holiday seasons. However, this could be a false-positive result (type I error) due to multiple hypothesis testing. The current study conducted a total of 10 hypothesis tests (one overall analysis, six stratified analyses, and three subgroup analyses) for each mortality outcome. According to the Bonferroni correction method, the p-value of hypothesis testing results should be less than 0.005 (0.05/10) to mitigate the risk of type I error.

Over the past two decades, several studies have investigated the association between weekend admissions and mortality risk in patients with AMI^2,3,20–25^. Furthermore, meta-analyses have summarized that weekend admission may be associated with an increased mortality risk in patients with AMI^7,26–31^. Previous findings have suggested that reduced staffing is a major driver of the increased mortality risk for weekend admissions; staff shortage might be more severe during the consecutive holiday seasons, causing an elevated mortality risk during the holiday season^32,33^. In a previous study reported by Liu et al.^34^ they found that admission on weekends did not significantly increase either in-hospital mortality or 1-year cumulative mortality compare to those admission on weekdays and this finding is comparable to ours. However, in Liu’s study, the authors defined “weekends” as all weekends plus national holidays. Our study specifically defined “weekends” as all weekends and “holiday season” as the Chinese New Year, a regular consecutive holiday period lasting 4 days (or more). In Taiwan, the operation of hospitals during weekends is quite different from this holiday season. We additionally found that admission on the holiday season did not increase the risk of in-hospital mortality or 7-day mortality compared to admission on weekends.

The age-sex standardized 30-day mortality rate (4.9%) for patients with AMI aged 45 years and above in our patients during the study period is very similar to that (4.7%) of the 25th percentile of a matched cohort reported by the Organization for Economic Cooperation and Development (OECD) database statistics from 2013 to 2020 across 38 member countries^35^. The relatively low incidence of mortality in Taiwan suggests that the services provided during holidays/weekends are equally good as those treated during weekdays. Our finding regarding the null effects of admission during holidays or weekends on the mortality risk in AMI patients is relevant to the health services and healthcare quality, which appear to be similarly good regardless of the admission time. In Taiwan, the health service program with various pre-hospital and in-hospital approaches has been endeavoring to reduce the onset-to-door and door-to-ballon times for these patients^36–39^. For example, many hospitals have established a specialized team to rapidly diagnose and treat AMI patients^40^. When needed, these pre-hospital and in-hospital approaches are activated not only on weekdays but also during holidays and weekends. These endeavors are supported by the healthcare system in Taiwan, which is known for its comprehensive coverage, high accessibility, and good quality of care^13,41^. In Taiwan, healthcare professionals who work on holidays or weekends can be due to mandatory rotations or working extra hours. In most, if not all, hospitals, healthcare professionals get overtime for working after the usual time or shift, although the compensation package may vary among hospitals. This policy ensures a high quality of healthcare during holidays and weekends.

The current study observed that among patients admitted for AMI and undergoing PCI, a higher proportion of transferred patients were in the holiday season group compared to the weekday group (15.9% vs. 11.7%, *P* < 0.001). While some previous studies speculated that transfers might prolong the time interval from symptom onset to PCI, recent research suggested that AMI patients who underwent inter-hospital transfer do not have a higher mortality risk. This may be attributed to the fact that transfers can provide patients to access to better healthcare resources^42^.

Since AMI is a prevalent disease worldwide, appropriate approaches are required to manage patients, particularly during the long holiday seasons. Previous studies have recommended that the excessive mortality risk related to weekend admissions could be reduced by maintaining nurse and physician staffing at the same level as that during weekdays; this approach may also work for the holiday seasons. However, insisting on additional duty hours during the holiday seasons or weekends is not a friendly strategy for healthcare workers^2,22^. Hence, there might need more coordination between healthcare providers, not only within the agency but also inter-hospital. However, the enhancement of healthcare quality cannot solely rely on efforts in hospitals, but also necessitates support from public health policies. Through this nationwide database study, we compared the differences in short- and medium -term mortality rates among patients with AMI, offering real-world evidence for future hospital management and public health policies.

Some limitations of present study should be considered. First, this study was conducted using a population from a single nation in Asia. Thus, the findings may not be suitable for inference to other countries because of different demographic characteristics, geographical features, healthcare systems, or emergency medical services. Second, some important clinical assessments, such as symptom presentation time, electrocardiogram results, troponin level, door-to-balloon time, left ventricular ejection fraction, and localization of myocardial infarction, could not be obtained from the NHIRD. Third, the current study did not include other national holidays (such as January 1 and February 28) in the holiday season group because these national holidays usually last for only one day. The operation of hospitals during the Lunar New Year holiday season (4 to 9 consecutive days) is very different from that of one-day national holidays. The last, some lifestyle-related factors, such as physical activity, alcohol use, and emotional stress, could not be used to adjust into the regression model. These factors were potential unmeasured confounders and may affect the study results^24,43,44^.

The current study found that the initial admission periods for patients with AMI receiving PCI are not associated with mortality outcomes, possibly due to a relatively small effect size. We conducted a post-hoc power calculation using STATA software (version 17). If the samples from this study are matched and set with a type I error of 0.05 and a power of 0.80, we can correctly detect a OR above 1.29. In plain language, the increased risk of mortality for AMI patients during hospitalization on holidays compared to weekdays was not greater than 29%.

In conclusion, this nationwide population-based cohort study revealed that in-hospital mortality and 7-day mortality were not higher among patients with AMI receiving PCI admitted during the holiday season compared with those admitted during weekdays and weekends.

### Supplementary Information

Supplementary Information.

## Data Availability

The datasets generated and/or analyzed during the current study are not publicly available due the privacy protection regulation of Health and Welfare Data Centre, Ministry of Health and Welfare, Taiwan but are available from the corresponding author on reasonable request. Researchers wishing to access this dataset can submit a formal application to the Taiwan Ministry of Health and Welfare (No. 488, Sec. 6, Zhongxiao E Rd., Nangang District, Taipei City 115, Taiwan; https://dep.mohw.gov.tw/DOS/cp-2516-59203-113.html) to request access.

## Abbreviations

AMI: Acute myocardial infarction
NHIRD: National Health Insurance Research Database
HWDC: Health and Welfare Data Center
ICD-9-CM: International Classification of Diseases, Ninth Revision, Clinical Modification
ICD-10-CM: International Classification of Diseases, Tenth Revision, Clinical Modification
STEMI: ST-elevation myocardial infarction
NSTEMI: Non-ST-elevation myocardial infarction
SMD: Standardized mean difference
aOR: Adjusted odds ratio
CI: Confidence interval
PCI: Percutaneous coronary intervention
CABG: Coronary artery bypass graft
ICU: Intensive care unit

## References

[1] GBD 2017 Disease and Injury Incidence and Prevalence Collaborators (2018). Global, regional, and national incidence, prevalence, and years lived with disability for 354 diseases and injuries for 195 countries and territories, 1990–2017: A systematic analysis for the Global Burden of Disease Study 2017. Lancet.

[2] Bell CM, Redelmeier DA (2001). Mortality among patients admitted to hospitals on weekends as compared with weekdays. N. Engl. J. Med..

[3] Kostis WJ (2007). Weekend versus weekday admission and mortality from myocardial infarction. N. Engl. J. Med..

[4] LaBounty T (2006). The impact of time and day on the presentation of acute coronary syndromes. Clin. Cardiol..

[5] Kim HJ (2015). The effect of admission at weekends on clinical outcomes in patients with non-ST-segment elevation acute coronary syndrome and its contributing factors. J. Korean Med. Sci..

[6] Maggs F, Mallet M (2010). Mortality in out-of-hours emergency medical admissions: More than just a weekend effect. J. R. Coll. Physicians.

[7] Sorita A (2014). Off-hour presentation and outcomes in patients with acute myocardial infarction: Systematic review and meta-analysis. BMJ.

[8] Eindhoven DC (2018). Mortality differences in acute myocardial infarction patients in the Netherlands: The weekend-effect. Am. Heart J..

[9] Lai CL, Kuo RN, Wang TC, Chan KA (2021). Mortality of major cardiovascular emergencies among patients admitted to hospitals on weekends as compared with weekdays in Taiwan. BMC Health Serv. Res..

[10] Tang L (2017). Effect of Chinese national holidays and weekends versus weekday admission on clinical outcomes in patients with STEMI undergoing primary PCI. J. Geriatr. Cardiol..

[11] Lin X (2020). Holiday and weekend effects on mortality for acute myocardial infarction in Shanxi, China: A cross-sectional study. Int. J. Public Health.

[12] Nicholls SG, Langan SM, Sorensen HT, Petersen I, Benchimol EI (2016). The RECORD reporting guidelines: Meeting the methodological and ethical demands of transparency in research using routinely-collected health data. Clin. Epidemiol..

[13] Hsieh CY (2019). Taiwan's national health insurance research database: Past and future. Clin. Epidemiol..

[14] Cheng CL (2014). Validation of acute myocardial infarction cases in the national health insurance research database in Taiwan. J. Epidemiol..

[15] Shelton SK (2018). Validation of an ICD code for accurately identifying emergency department patients who suffer an out-of-hospital cardiac arrest. Resuscitation.

[16] Wittwer MR (2021). Overcoming challenges of establishing a hospital-based out-of-hospital cardiac arrest registry: Accuracy of case identification using administrative data and clinical registries. Resusc Plus.

[17] Quan H (2005). Coding algorithms for defining comorbidities in ICD-9-CM and ICD-10 administrative data. Med. Care.

[18] Gandhi S (2013). Calcium-channel blocker-clarithromycin drug interactions and acute kidney injury. JAMA.

[19] Heinze G, Juni P (2011). An overview of the objectives of and the approaches to propensity score analyses. Eur. Heart J..

[20] Casella G (2011). Off-hour primary percutaneous coronary angioplasty does not affect outcome of patients with ST-Segment elevation acute myocardial infarction treated within a regional network for reperfusion: The REAL (Registro Regionale Angioplastiche dell'Emilia-Romagna) registry. JACC Cardiovasc. Interv..

[21] de Boer SP (2012). Primary PCI during off-hours is not related to increased mortality. Eur. Heart J. Acute Cardiovasc. Care.

[22] de Cordova PB, Johansen ML, Martinez ME, Cimiotti JP (2017). Emergency department weekend presentation and mortality in patients with acute myocardial infarction. Nurs. Res..

[23] Harhash AA (2021). Comparison of outcomes among patients with cardiogenic shock admitted on weekends versus weekdays. Am. J. Cardiol..

[24] Kim SS (2014). Impact of patients' arrival time on the care and in-hospital mortality in patients with non-ST-elevation myocardial infarction. Am. J. Cardiol..

[25] Oh S, Hyun DY, Cho KH, Kim JH, Jeong MH (2021). Long-term outcomes in ST-elevation myocardial infarction patients treated according to hospital visit time. Korean J. Intern. Med..

[26] Enezate TH (2017). Comparison of outcomes of ST-elevation myocardial infarction treated by percutaneous coronary intervention during off-hours versus on-hours. Am. J. Cardiol..

[27] Kwok CS (2019). Weekend effect in acute coronary syndrome: A meta-analysis of observational studies. Eur. Heart J. Acute Cardiovasc. Care.

[28] Wang B (2017). Off-hours presentation is associated with short-term mortality but not with long-term mortality in patients with ST-segment elevation myocardial infarction: A meta-analysis. PLoS ONE.

[29] Wang X (2015). Is there an association between time of admission and in-hospital mortality in patients with non-ST-elevation myocardial infarction? A meta-analysis. Sci. Rep..

[30] Yu YY, Zhao BW, Ma L, Dai XC (2021). Association between out-of-hour admission and short- and long-term mortality in acute myocardial infarction: A systematic review and meta-analysis. Front. Cardiovasc. Med..

[31] Zhou Y (2016). Off-hour admission and mortality risk for 28 specific diseases: A systematic review and meta-analysis of 251 cohorts. J. Am. Heart Assoc..

[32] Freemantle N (2015). Increased mortality associated with weekend hospital admission: A case for expanded seven day services?. BMJ.

[33] Ozdemir BA (2016). Mortality of emergency general surgical patients and associations with hospital structures and processes. Br. J. Anaesth..

[34] Liu SF (2023). Mortality among acute myocardial infarction patients admitted to hospitals on weekends as compared with weekdays in Taiwan. Sci. Rep..

[35] The Organisation for Economic Co-operation and Development (OECD). *OECD Statistics*. https://stats.oecd.org/. Accessed 1 Mar 2024.

[36] Li YH (2019). Changing practice pattern of acute coronary syndromes in Taiwan from 2008 to 2015. Acta Cardiol. Sin..

[37] Lu TH (2015). Characteristics of early and late adopting hospitals providing percutaneous coronary intervention in Taiwan. J. Am. Heart Assoc..

[38] Yin WH (2016). The temporal trends of incidence, treatment, and in-hospital mortality of acute myocardial infarction over 15years in a Taiwanese population. Int. J. Cardiol..

[39] Yu SH, Shih HM, Chang SS, Chen WK, Li CY (2019). Social media communication shorten door-to-balloon time in patients with ST-elevation myocardial infarction. Medicine.

[40] Lin J-F (2010). Data feedback reduces door-to-balloon time in patients with ST-elevation myocardial infarction undergoing primary percutaneous coronary intervention. Heart Vessels.

[41] Ho Chan WS (2010). Taiwan's healthcare report 2010. EPMA J..

[42] Mueller S, Zheng J, Orav EJ, Schnipper JL (2019). Inter-hospital transfer and patient outcomes: A retrospective cohort study. BMJ Qual. Saf..

[43] Edahiro R (2014). Association of lifestyle-related factors with circadian onset patterns of acute myocardial infarction: A prospective observational study in Japan. BMJ Open.

[44] Walker AS (2017). Mortality risks associated with emergency admissions during weekends and public holidays: An analysis of electronic health records. Lancet.

