# Supplementary material for: Association between holiday and weekend admissions and mortality outcomes among patients with acute myocardial infarction receiving percutaneous coronary intervention in Taiwan
Source: Sci Rep. 2024 Apr 17;14:8892. doi: 10.1038/s41598-024-59571-w (PMC11023946; doi:10.1038/s41598-024-59571-w)
Supplement: Supplementary file 1 — Supplementary Information. [file 41598_2024_59571_MOESM1_ESM.pdf]

Table S1. Codes for medical services

Order name	Codes
PCI	Taiwan NHI claim codes:<br>18020B, 18021B, 33076A, 33076B, 33077A, 33077B, 33078A, 33078B, 97501K, 97502A, 97503B, 97506K, 97507A, 97508B, 97511K, 97512A, 97513B, 97516K, 97517A, 97518B, 97521K, 97522A, 97523B<br>ICD-9-CM procedure codes:<br>00.66, 17.55, 36.01, 36.02, 36.05, 36.06, 36.07<br>ICD-10-CM procedure codes:<br>02C03, 02H03
ICU	Taiwan NHI claim codes:<br>02011K, 02012A, 02013B, 03010E, 03011F, 03012G, 03017A, 03041A, 03041A, 03047E, 03048F, 03049G, 05151B, 05152B, P6301B
CABG	Taiwan NHI claim codes:<br>68023A, 68023B, 68024A, 68024B, 68025A, 68025B, 97901K, 97902A, 97903B, 97906K, 97907A, 97908B, 97911K, 97912A, 97913B, 97916K, 97917A, 97918B<br>ICD-9-CM procedure codes:<br>36.1, 36.2<br>ICD-10-CM procedure codes:<br>0210, 0211, 0212, 0213

Abbreviations: PCI, Percutaneous coronary intervention; ICU, intensive care unit; CABG, coronary artery bypass graft; NHI, National Health Insurance; ICD-9-CM, International Classification of Diseases, Ninth Revision, Clinical Modification; ICD-10-CM, International Classification of Diseases, Tenth Revision, Clinical Modification.

Table S2. Dates of Chinese New Year holiday seasons in Taiwan

Year	Chinese New Year holiday seasons	Chinese New Year holiday seasons extended with a nearby weekend
2013	Feb 9–Feb 12	Feb 9–Feb 12
2014	Jan 30–Feb 2	Jan 30–Feb 2
2015	Feb 18–Feb 21	Feb 18–Feb 22
2016	Feb 7–Feb 10	Feb 7–Feb 10
2017	Jan 27–Jan 30	Jan 27–Jan 30
2018	Feb 15–Feb 18	Feb 15–Feb 20
2019	Feb 04–Feb 07	Feb 02–Feb 10
2020	Jan 24–Feb 27	Jan 24–Jan 27

Abbreviations: Jan, January; Feb, February.

Table S3. Comparison of baseline characteristics between patients admitted during holiday seasons, weekends, and weekdays using standardized mean difference

Variable	Standardized mean difference		
	Holiday season vs. weekday	Weekend vs. weekday	Holiday season vs. weekend
Age* (yeas)	0.012	0.010	0.001
< 65	0.022	0.002	0.020
≥ 65	0.022	0.002	0.020
Sex			
Male	0.035	0.009	0.026
Female	0.035	0.009	0.026
Insurance premium level			
Financially dependent	0.020	0.020	0.040
15,840–24,999	0.071	0.010	0.081
25,000–44,999	0.035	0.016	0.019
≥ 45,000	0.040	0.003	0.043
Hospital level			
Medical center	0.105	0.034	0.070
Regional hospital	0.105	0.034	0.070
Hospital area location			
North	0.053	0.006	0.059
Central	0.012	0.017	0.005
South	0.040	0.015	0.055
East	0.011	0.024	0.035
Physician type			
Cardiologist	0.027	0.003	0.023
Cardiothoracic surgeon	0.023	0.009	0.032
Other	0.013	0.013	0.000
AMI type			
STEMI	0.059	0.028	0.030
NSTEMI	0.059	0.028	0.030
Transferred-in	0.122	0.094	0.028
OHCA	0.019	0.038	0.018
CABG	0.030	0.010	0.020
ICU	0.037	0.062	0.025
Length of stay*	0.001	0.006	0.005
CCI score*	0.010	0.045	0.035
Comorbidities			
DM	0.017	0.035	0.019
HTN	0.046	0.004	0.042
COPD	0.032	0.019	0.050

Heart failure	0.062	0.030	0.032
CAD	0.060	0.027	0.033
CKD	0.000	0.063	0.063
Cirrhosis	0.041	0.000	0.041
Stroke	0.016	0.006	0.023
Dementia	0.006	0.029	0.023
Malignancy	0.000	0.005	0.005
Index year			
2013	0.113	0.013	0.126
2014	0.131	0.028	0.103
2015	0.006	0.009	0.003
2016	0.059	0.067	0.126
2017	0.061	0.018	0.079
2018	0.132	0.012	0.120
2019	0.264	0.092	0.354
2020	0.109	0.009	0.118

Abbreviations: AMI, acute myocardial infarction; STEMI, ST-elevation myocardial infarction; NSTEMI, non-ST-elevation myocardial infarction; OHCA, out-of-hospital cardiac arrest; CABG, coronary artery bypass graft; ICU, intensive care unit; CCI, Charlson comorbidity index; DM, diabetes mellitus; HTN, hypertension; COPD, chronic obstructive pulmonary disease; CAD, coronary arterial disease; CKD, chronic kidney disease.

Table S4. Number of AMI cases per day during the observation period

Year	Holiday season	Weekend	Weekday
2013	34	39	39
2014	37	39	44
2015	40	36	44
2016	46	37	50
2017	48	43	48
2018	50	44	47
2019	44	39	49
2020	43	43	48

Abbreviations: AMI, acute myocardial infarction.

Table S5. Mortality rates in patients with AMI during the holiday seasons, weekends, and weekdays stratified by hospital levels.

Mortality type	Date category	Medical center			Regional hospital			*P* value*
		N	Mortality events	Mortality rate (%)	N	Mortality events	Mortality rate (%)	
In-hospital mortality	Holiday season	827	49	5.9	902	70	7.8	0.132
	Weekend	2092	136	6.5	2633	161	6.1	0.587
	Weekday	6214	366	5.9	8369	553	6.6	0.078
7-day mortality	Holiday season	827	24	2.9	902	40	4.4	0.098
	Weekend	2092	71	3.4	2633	88	3.3	0.935
	Weekday	6214	151	2.4	8369	314	3.8	<.001

* The *P* values were obtained using chi-square test.

Abbreviations: AMI, acute myocardial infarction.

Table S6. Mortality rates in patients with AMI during the holiday seasons, weekends, and weekdays stratified by geographic regions.

Mortality type	Date category	North			Central			South			East			*P* value*
		N	Mortality events	Mortality rate (%)	N	Mortality events	Mortality rate (%)	N	Mortality events	Mortality rate (%)	N	Mortality events	Mortality rate (%)	
In-hospital mortality	Holiday season	705	57	8.1	372	23	6.2	595	36	6.1	57	3	5.3	0.432
	Weekend	2066	121	5.9	1027	73	7.1	1504	94	6.3	128	9	7.0	0.583
	Weekday	6336	391	6.2	3057	202	6.6	4745	298	6.3	445	28	6.3	0.880
7-day mortality	Holiday season	705	40	5.7	372	9	2.4	595	13	2.2	57	2	3.5	0.004
	Weekend	2066	63	3.0	1027	44	4.3	1504	48	3.2	128	4	3.1	0.337
	Weekday	6336	188	3.0	3057	116	3.8	4745	148	3.1	445	13	2.9	0.197

The geographic regions include northern, central, southern, and eastern Taiwan.

* The *P* values were obtained using chi-square test.

Abbreviations: AMI, acute myocardial infarction.

Figure S1. 7-day mortality risks in patients with AMI during the holiday seasons, weekends, and weekdays stratified by sex, age group, and AMI type

Subgroups	Comparison	7-day mortality	aOR (95% CI)	p
Male	Holiday vs weekday (ref.)		1.26 (0.89–1.74)	0.178
	Weekend vs weekday (ref.)		1.03 (0.81–1.30)	0.812
Female	Holiday vs weekday (ref.)		1.08 (0.63–1.75)	0.781
	Weekend vs weekday (ref.)		0.95 (0.68–1.31)	0.760
< 65 years	Holiday vs weekday (ref.)		0.82 (0.42–1.48)	0.536
	Weekend vs weekday (ref.)		0.91 (0.60–1.35)	0.643
≥ 65 years	Holiday vs weekday (ref.)		1.33 (0.96–1.81)	0.072
	Weekend vs weekday (ref.)		1.02 (0.82–1.26)	0.863
STEMI	Holiday vs weekday (ref.)		0.98 (0.63–1.47)	0.932
	Weekend vs weekday (ref.)		1.18 (0.91–1.53)	0.209
NSTEMI	Holiday vs weekday (ref.)		1.46 (0.98–2.10)	0.051
	Weekend vs weekday (ref.)		0.83 (0.62–1.11)	0.218

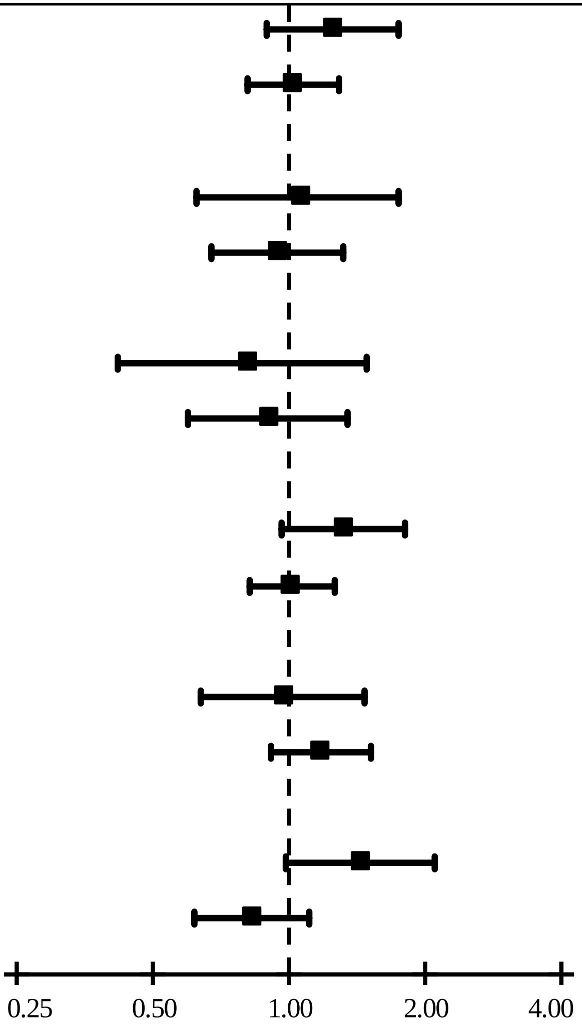

Abbreviations: AMI, acute myocardial infarction; aOR, adjusted odds ratio; CI, confidence interval; ref, reference group; STEMI, ST-elevation myocardial infarction; NSTEMI, non-ST-elevation myocardial infarction.

Figure S2. Sensitivity analysis for 7-day mortality risk after excluding patients underwent CABG, transferred, and OHCA

Excluding criteria	Comparison	7-day mortality	aOR (95% CI)	p
CABG	Holiday vs weekday (ref.)		1.17 (0.87–1.56)	0.280
	Weekend vs weekday (ref.)		1.01 (0.82–1.22)	0.949
Transferred	Holiday vs weekday (ref.)		1.22 (0.89–1.63)	0.203
	Weekend vs weekday (ref.)		0.97 (0.79–1.19)	0.778
OHCA	Holiday vs weekday (ref.)		1.24 (0.92–1.65)	0.148
	Weekend vs weekday (ref.)		0.99 (0.80–1.22)	0.945

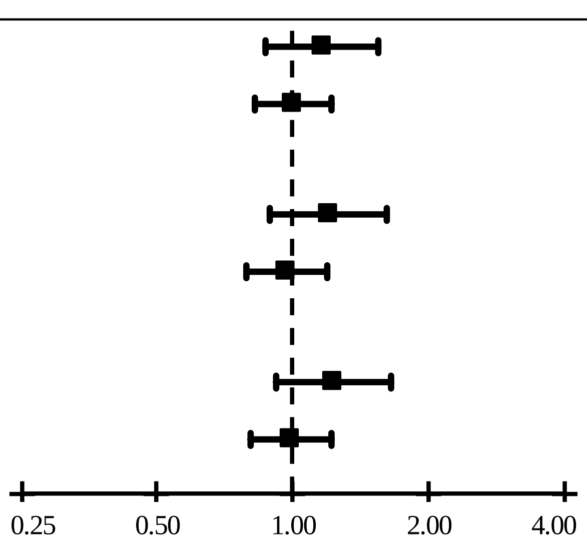

Abbreviations: aOR, adjusted odds ratio; CI, confidence interval; ref, reference group; CABG, coronary artery bypass graft; OHCA, out-of-hospital cardiac arrest.
